# PTSD and loss: preliminary findings from a territory-wide epidemiology study in Hong Kong

**DOI:** 10.3402/ejpt.v5.26517

**Published:** 2014-12-09

**Authors:** Kitty K. Wu

**Affiliations:** Kwai Chung Hospital, Hong Kong, People's Republic of China

**Keywords:** loss, grief, prevalence, trauma, social support, screening

## Abstract

**Background:**

The study examined the prevalence of trauma and posttraumatic stress disorder (PTSD) symptoms among community dwelling Chinese adults in Hong Kong. The relationship of traumatic life events (including loss) and mental health has been investigated.

**Methods:**

The sampling of the collaborative study (HKMMS: Hong Kong Mental Morbidity Survey) adopts a multi-stage stratification approach with the distribution of residential premises in different geographical districts and the relative proportion of private versus public housing units taken into consideration. In Phase I of this study, 4,644 adults were screened for PTSD with the Trauma Screening Questionnaire (TSQ) and Life Event Checklist (LEC), Beck's scales and CIS-R (Revised Clinical Interview Schedule). In Phase II of the study, clinical psychologists conducted the Structured Clinical Interview for DSM Disorders (SCID) for 92 participants (results not reported here).

**Results:**

Among Phase I participants, 65% reported traumatic experience (including 18% who reported personal experience of sudden death of significant others). Age and gender make a difference in traumatic experience. When compared to participants who reported no traumatic experience in the past, participants who reported to have personal experience of sudden death of significant others or other traumatic experiences were found to have higher TSQ scores, higher psychological distress, lower social support (PSS: Multidimensional Scale of Perceived Social Support), and lower life functioning (SOFAS: Social and Occupational Functioning Assessment Scale), *p*<0.001. Findings of hierarchical regression showed that type of trauma (i.e., loss, other trauma, or no trauma) contributed significantly to the prediction of all the mental health indices after demographic and social variables were controlled.

**Conclusions:**

Public education on the association of traumatic experience and psychological health, as well as the monitoring of mental health for at-risk individuals are suggested for early identification of people in need of mental health services.

